# Selective Electrochemical Production of Ethylene from Bicarbonate Solution

**DOI:** 10.1002/anie.202509975

**Published:** 2025-07-17

**Authors:** Behnam Nourmohammadi Khiarak, Gelson T. S. T. da Silva, Jackson Crane, Colin P. O'Brien, Michael R. Pepe, Christine M. Gabardo, Viktoria Golovanova, F. Pelayo García de Arquer, Cao‐Thang Dinh

**Affiliations:** ^1^ Department of Chemical Engineering Queen's University Kingston ON K7L 3N6 Canada; ^2^ Interdisciplinary Laboratory of Electrochemistry and Ceramics Department of Chemistry Federal University of Sao Carlos São Carlos SP 13565–905 Brazil; ^3^ Department of Mechanical and Materials Engineering Queen's University Kingston Ontario K7P 3N6 Canada; ^4^ CERT Systems Inc. Toronto Ontario M6N 2J1 Canada; ^5^ ICFO – Institut de Ciències Fotòniques The Barcelona Institute of Science and Technology Barcelona 08860 Spain

**Keywords:** Cu oxide derive catalysts, Direct bicarbonate electroreduction, Electrochemistry, Ethylene production, Porosity and thickness effect

## Abstract

Carbon dioxide (CO_2_) electroreduction directly from a reactive carbon solution (e.g., (bi)carbonate) provides a promising approach for integrating CO_2_ capture and conversion. Compared to CO_2_ conversion in gas‐fed systems, this system typically suffers from low Faradaic efficiency (FE), especially for multicarbon (C_2+_) products. Here, we report an engineered material structuring to selectively produce C_2+_ products directly from a N_2_‐saturated bicarbonate solution. Multiphysics modeling studies reveal the critical role of local current density distribution and the spatio‐selective evolution of C_2+_ products, which is favored in thinner catalysts (240 µm thickness). By jointly tailoring catalyst configuration and mass transport in bicarbonate electroreduction, adjusting the thickness, porosity, and surface oxidation of copper (Cu) mesh catalysts, as well as catholyte composition, we achieved a maximum C_2_H_4_ FE of 39% and total C_2+_ FE over 55% at 150 mA cm^−2^ with a 240 µm thick Cu mesh. The system is also stable for over 160 h at 100 mA cm^−2^ with maintained C_2_H_4_ FE over 20%. Our electrolysis system converts bicarbonate to C_2+_ with over 90% CO_2_ utilization efficiency, reducing regeneration and separation costs. Optimizing catalyst pore structure, and copper surface oxide is a key to maximizing C_2_H_4_ production from bicarbonate solutions.

## Graphical abstract

We report direct electroreduction of bicarbonate to C_2+_ products using Cu mesh catalysts. The effect of mesh thickness, pore size, catholyte composition, and thermal treatment was systematically studied. Our finding shows that thinner Cu mesh showed enhanced C_2+_ selectivity due to improved mass transport, while heat‐treated meshes form oxide‐derived surfaces that shift selectivity from hydrogen evolution to efficient eCO_2_R.

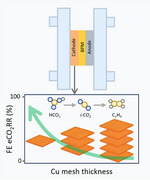

## Introduction

Electrochemical CO_2_ reduction (eCO_2_R) has emerged as a promising technology for converting CO_2_ into valuable chemicals and fuels, offering a potential solution for reducing greenhouse gas emissions.^[^ ^1^, ^2^, ^3^ ^]^ C_2_H_4_ is an attractive product of eCO_2_R, due to its large global market of ∼US $240B, and its wide use in industrial applications, including the production of plastics, resins, and fibers.^[^ ^4^, ^5^, ^6^ ^]^

In many studies focusing on eCO_2_R, electrolyzers typically require high‐purity CO_2_ supplied at elevated pressures. This process requires additional energetic steps to generate a pure gas‐phase CO_2_ stream and separation. Direct electroreduction of capture solution (alkaline capture solutions) could bypass the energy‐intensive step of purifying CO_2_ stream from the captured medium.^[^ ^7^, ^8^ ^]^ eCO_2_R systems have several common components: a cathode, an anode, and a separator. The cathode is where CO_2_ reduction takes place, the anode is where oxidizing reactions occur (typically oxygen evolution), and the separator isolates the two reactions, and is typically a membrane which selectively transports cations or anions. Within this common architecture, there are several different configurations. In gas phase conversion cells, a gas diffusion layer at the cathode enables the diffusion of gas‐phase CO_2_ to the catalyst, where a triple‐phase boundary (gas‐phase CO_2_, liquid phase electrolyte, and solid phase catalyst) facilitates the reduction reaction.^[^ ^9^, ^10^ ^]^ In a dissolved CO_2_ aqueous conversion system, pre‐dissolved CO_2_ in catholyte is supplied to the cathode surface for the purpose of reduction reaction.^[^ ^11^ ^]^ Finally, in direct (bi)carbonate‐based aqueous conversion systems, CO_2_ is generated in situ to provide dissolved and gas‐phase CO_2_ to the cathode surface for subsequent conversion. The local aqueous bicarbonate (HCO_3_^−^) can react with proton (H^+^) ions coming from membrane, specifically referring to H^+^ ions generated from water splitting within the bipolar membrane (BPM), which then diffuse through the cation exchange layer of BPM to the cathode side.^[^ ^12^ ^]^ This is resulting in the in situ formation of CO_2_ [referred to as *i‐CO_2_*, according to equation 1 (Equation 1)].

(1) $${\text{HCO}}_{3^{-}\left(\mathrm{aq}\right)}+H_{\left(\mathrm{aq}\right)}^{+}\to i-CO_{2(g)}+H_{2}O_{\left(l\right)}$$

subsequently, *i‐CO_2_* can then undergo electrochemical reduction to yield eCO_2_R products.^[^ ^7^ ^]^

The efficient generation of *i‐CO_2_* requires sufficient H^+^, which are typically provided from an acidic anodic environment via a cation exchange membrane (CEM) or generated from water dissociation within a BPM.

In direct bicarbonate electroreduction systems using a BPM, Lees et al., ^[^ ^7^ ^]^ demonstrated high FE of over 60% for the production of carbon monoxide (CO) and formate at current densities exceeding 100 mA cm^−2^, and an FE of 34% for methane (CH_4_) at a partial current density of 120 mA cm^−2^. The production of C_2+_ compounds, such as C_2_H_4_, as primary products in bicarbonate‐based systems has received relatively little attention due to the challenges associated with their electrosynthesis. Most recently, Wang et.al., ^[^ ^13^ ^]^ have shown the significance of the local catalyst microenvironment in enhancing bicarbonate conversion to C_2_H_4_, achieving 31% of FE to C_2_H_4_ at a low current density of 100 mA cm^−2^. Yet, the bicarbonate conversion systems suffer from limited local CO_2_ availability and low local catalyst surface alkalinity, both of which strongly influence the FE toward C_2+_ products in CO_2_ electrolysis. Also, the recent research suggests that achieving improved FE to C_2+_ products requires a balance between mass transport and catalytic activity.^[^ ^14^ ^]^

Cu shows promise in C_2_H_4_ production via eCO_2_R. Extensive research has focused on enhancing C_2_H_4_ FE in CO_2_‐fed electrolyzers through Cu‐based catalyst design and local environment modification. Optimizing pH levels, electrolyte composition, and surface properties fine‐tunes the local environment to boost C_2_H_4_ production, reduce byproducts, and control reaction kinetics and surface morphology.^[^ ^15^, ^16^ ^]^

Oxide‐derived Cu catalysts improve FE toward C_2+_ products by facilitating local environment, such as high local pH, CO_2_ molecule adsorption, and reaction intermediate formation, favoring C–C coupling and CO coverage at cathode surface.^[^ ^17^, ^18^ ^]^ The synergy between oxide/metallic phases in Cu‐based electrocatalysts enhances CO_2_ activation and *CO dimerization, improving thermodynamics and kinetics for C_2_ production.^[^ ^19^, ^20^, ^21^ ^]^ Alkali metal cations, like K^+^ and Cs^+^_,_ affect FE by influencing intermediates' adsorption on cathodic surfaces, leading to more positive potential values for C_2_ products. Anions (Cl^−^, Br^−^, I^−^) in the catholyte adsorb on the surface, increasing the negative charge on Cu and promoting CO protonation for C_2_ products.^[^ ^22^, ^23^, ^24^ ^]^ However, replicating successful gas‐fed reactor conditions in bicarbonate‐fed reactors poses challenges due to CO_2_ availability, lower bulk pH, CO_2_ and bicarbonate mass transport limitation, and catalyst morphology constraints.

Here, we pursue C_2+_ product generation from bicarbonate solution by simultaneously optimizing the local environment, CO_2_ availability, and catalyst thickness and porosity, together with Cu oxidation state.

We control the local pH and concentration of carbon species [e.g., CO_3_^2−^, *i‐CO*_2(g)_, CO_2(aq)_] by varying electrolyte concentration and ionic strength. CO_2_ availability on the catalyst surface can be augmented through tuning the porosity and thickness of electrodes. The catalyst oxidation state was adjusted by heat treatment under ambient condition.

We observed that thinner catalysts favor bicarbonate conversion into C_2_H_4_. We reasoned that efficient electrolysis of bicarbonate to C_2_H_4_ necessitates a thin and porous electrode to facilitate the transport of aqueous bicarbonate solutions to the catalyst/membrane interface, where *i‐CO_2_* can be generated. Cu catalyst oxidation state also appears to contribute significantly to the catalytic performance.

To offer physical insights into the mechanism, we have conducted multiphysics modeling, which suggests that catalyst with a thickness of 240 µm exhibits improved performance due to highly inhomogeneous local current density within the catalyst layer. Specifically, the majority of C_2+_ evolution occurs near the catalyst edges, at the interface between the membrane and the catalyst electrode layer.

In our study, we achieved a FE for C_2_H_4_ production of approximately 39% at 150 mA cm^−2^ using a microscale Cu mesh catalyst with a thickness of 240 µm. We demonstrated a total C_2+_ production of over 55% at 150 mA cm^−2^ and a high CO_2_ utilization efficiency of over 90% at all tested current density ranges. Through a regeneration strategy with alternating “on” (300 s) and “off” (200 s) electrolysis segments, we maintained high selectivity toward C_2_H_4_ of over 20% for over 160 h at a current density of 100 mA cm^−2^.

## Results and Discussion

### The Electrolyser and System Configuration

**Figure 1:**
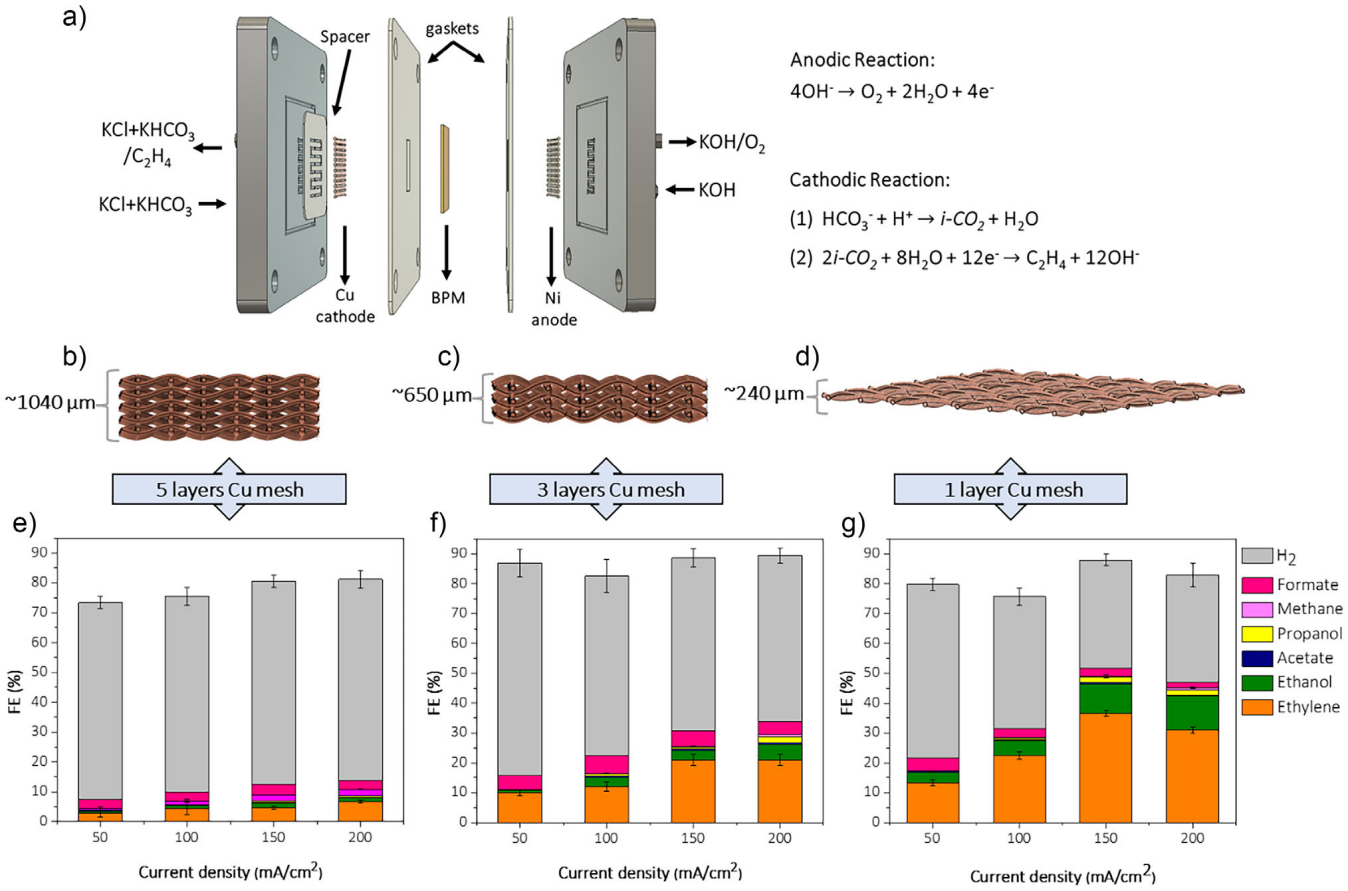
a) Schematic representation of the bicarbonate electrolyzer used in this work to produce C_2+_ from a reactive carbon solution (KHCO_3_), b)–d) Schematic representation of catalysts with different thicknesses (Cu mesh 200*200 PPI); (b) ∼1040 µm, (c) ∼650 µm, and (d) ∼240 µm, and e)–g) the FE of products over the Cu mesh with different thickness.

The bicarbonate electrolyzer used in this study was a membrane electrode assembly (MEA) electrochemical cell with an active area of 2 cm^2^ (Figure 1a). A BPM operating under reverse bias was used to separate cathode and anode in the MEA cell. The bicarbonate solution was purged with N_2_ for 10 minutes prior to the reaction and continuously purged during eCO_2_R. During the CO_2_ reduction reaction, water is dissociated into H^+^, which migrate through the cation exchange layer to the cathode, and hydroxyl ions (OH^−^), which migrate through the anion exchange layer to the anode (Figure 1a). At the nickel (Ni) foam anode, OH^−^ ions are oxidized to form oxygen (O_2_) and H_2_O. Meanwhile, the H^+^ ions at the cation exchange layer (CEL)/cathode interface react with HCO_3_^−^ to generate *i‐CO_2_* (Equation 1), which is then reduced to hydrocarbons on the surface of the cathode

### Catalyst Structure Effect

It is well documented that modulating the electrode surface microenvironment can help to enhance electrocatalytic eCO_2_RR efficiency. Surface roughening—such as through oxidation—can increase the density of active sites and expose specific facets, which locally concentrates CO_2_ and improves reaction performance.^[^ ^25^, ^26^, ^27^, ^28^ ^]^ Additionally, free‐standing porous metals have demonstrated the feasibility of utilizing the entire cathode in catalytic systems, offering advantages in terms of structural stability and electrochemical activity. In our experimental endeavors, we initially examined the performance of an untreated Cu mesh (Figure S1a) in 1 M KHCO_3_ as a potential cathode material for direct bicarbonate conversion. However, our findings revealed significantly low C_2_H_4_ FE of less than 2% (Figure S1a). This suboptimal performance can be attributed to poor active sites (not selective for eCO_2_RR) and local environment to stabilize key intermediates like CO adsorption and CO–CO for the formation of C_2+_ products, which limit their effectiveness in absorbing and reducing CO_2_ molecules. Consequently, we turned our attention to testing oxide‐derived Cu materials, aiming to exploit their unique properties and potentially enhance the electrocatalytic performance of the system.

Next, we conducted tests on a Cu mesh that was pretreated with heat treatment (HT) at 340 °C for 3 h in the air atmosphere. As the results show (Figure S1b), the HT Cu mesh exhibited an 8.5% FE for C_2_H_4_ production at 150 mA cm^−2^ in 1 M KHCO_3_ catholyte, while the untreated Cu mesh showed an FE of approximately 1% for C_2_H_4_ at the same current density. This corresponds to 8.5 times increase in FE for C_2_H_4_ production following the HT of the Cu mesh at 340 °C compared to the untreated Cu mesh. Although the HT Cu mesh exhibited an 8.5% FE for C_2_H_4_ at 150 mA cm^−2^, this performance is relatively low compared to reported performance for direct bicarbonate conversion (Table S1). It was hypothesized that the elevated concentration of KHCO_3_ might buffer the local catalyst surface, leading to a significant decrease in C_2_H_4_ FE. Given that the H^+^ flux originated from the BPM in proximity to the catalyst surface, we decreased the KHCO_3_ concentration to lower its buffer effect and introduced KCl to leverage the shielding effect of K^+^ ions and mitigate the hydrogen evolution reaction (HER) interference. So, we have chosen 0.1 M KHCO_3_ + 0.9 M KCl catholyte to test our hypothesis.

We investigated the effect of catalyst structure on direct bicarbonate electroreduction to C_2_H_4_ using heat‐treated Cu‐based samples. We tested three different catalyst configurations: i) five layers of stacked Cu meshes with a total thickness of 1040 µm, ii) three layers of stacked Cu meshes with a total thickness of 650 µm, and iii) a single layer of Cu mesh with a total thickness of 240 µm. All Cu meshes used in this study had a porosity of 200 Pores Per Inch (PPI) (Figure 1b–d). The electrochemical reduction in 0.1 M KHCO_3_ + 0.9 M KCl solution was tested on each of the electrode materials at various current densities, for identical HT of 340 °C for 3 h under ambient condition. The five layers of stacked Cu meshes with a thickness of 1040 µm (Figure 1b) produced almost entirely H_2_ under all screened current densities, with a small amount of C_2_H_4_ (∼9%) at a current density of 200 mA cm^−2^ with a less than 5% FE for ethanol and total C_2+_ FE < 12% (Figure 1e). A cathode with three stacked layers of Cu meshes, having a total thickness of around 650 µm (Figure 1c) demonstrated improved performance, with a maximum C_2_H_4_ FE of ∼20% at 200 mA cm^−2^ and total C_2+_ FE of ∼28.5% (Figure 1f). Reducing the number of layers of Cu mesh to a single layer (Figure 1d), a maximum C_2_H_4_ FE of 36% at 150 mA cm^−2^ was achieved with total C_2+_ FE ∼50% (Figure 1g). CH_4_ is another product observed with an FE in the range of 0.5–6% at all applied current densities for all samples (Figure 1e–g). The full cell voltage for single‐layer Cu mesh substrate was 3.8 V at the current density of 50 mA cm^−2^ and increased to 4.2, 4.5, and 4.95 V at the current densities of 100, 150, and 200 mA cm^−2^, respectively (Figure S2). The time dependencies of the full cell voltage for different cathode configurations are shown in Figure S2a‐c.

As demonstrated in the above observations, each of the cathode configurations with different porosity and thickness exhibited distinct activities in bicarbonate conversion (Figure 1b–g). We reason that the single‐layer Cu mesh facilitates more efficient mass transport compared to the stacked Cu meshes. Additionally, as the catalyst thickness increases, the CO_2_ availability at the flow plate‐catalyst interface decreases due to the longer diffusion path. This depletion of *i‐CO_2_* results in higher resistance and overpotential for CO_2_ conversion at the outer layers, which, in turn, increases the HER activity and reduces the selectivity for C_2_H_4_.

Furthermore, we reason that a thicker catalyst would promote HER at sites far from the catalyst‐membrane interface. This is attributed to the reduced CO_2_ availability with increasing distance from the BPM. Previous work has suggested that the majority of CO_2_ conversion takes place within 50 to 100 µm from the catalyst‐membrane interface.^[^ ^29^ ^]^ Therefore, our experiments with substantially thicker catalysts may extend to greater distances from BPM, where HER becomes dominant. Next, we have employed multiphysics modeling to explore local pH and CO_2_ availability on the catalyst surface to further support our hypothesis regarding lower C_2+_ product FE for thicker cathodes.

It should be noted that the CO_2_ conversion efficiency of the HT Cu catalysts improve over time. As shown in Figure S2d, initially the catalyst demonstrates ∼30% FE after 10 min of the reaction. Over time, the C_2_H_4_ FE reaches a maximum (35.5%), before declining to 28% within 3000 s of the reaction.

### Modeling Reaction Environment and CO_2_ Availability

**Figure 2:**
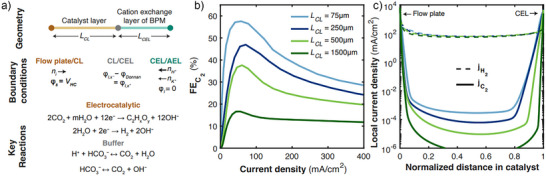
a) Overview of modelled geometry, boundary conditions, and key reactions within system, b) modelled C_2_ FE as a function of current density for catalysts of varying width, c) local current density within catalyst domain for HER (dashed lines) and C_2_ evolution (solid lines) for the same catalyst widths as in (b) for a half‐cell potential of −1.7 V (∼100 mA cm^−2^ total current density for all widths).

To investigate our hypothesis regarding the relative catalyst thickness dependency on C_2_ productivity, we performed COMSOL Multiphysics modeling of chemical species generation, consumption, and mass diffusion in different configurations to identify an optimum cathode configuration. The modeling considered the thickness of the cathode and membrane, electrocatalytic and bicarbonate buffer reactions, electro migrative and diffusive transport phenomena, and CO_2_ phase transfer (Figure 2a).^[^ ^30^, ^31^ ^]^

We hypothesized that a major contributor to the difference in performance of Cu mesh in different stacks are their thicknesses (240 to 1040 µm). Several simulations were performed with varying catalyst layer thickness. As the catalyst layer became thinner, the predicted C_2_ FE increased, consistent across all current densities (Figure 2b), and aligning with experimental trends. As the modeling suggests and previous studies have shown, the *i‐CO_2_* concentration is high near the catalyst‐membrane interface and also at the flow plate‐catalyst interface (Figure S3). The low CO_2_ concentration in the center of the catalyst domain leads to low partial C_2_ current density in that region (Figure 2c). Meanwhile, the HER rates remain relatively uniform throughout the domain. Therefore, as the catalyst domain becomes thicker, the relative contribution of HER becomes more pronounced, leading to lower overall C_2_ FE for the thicker catalysts.

### Effect of Thermal Treatment

To enhance the FE for bicarbonate conversion and concurrently mitigate HER, we aimed to restructure the surface characteristics of the copper catalyst optimizing heat treatment parameters such as temperature and time.

**Figure 3:**
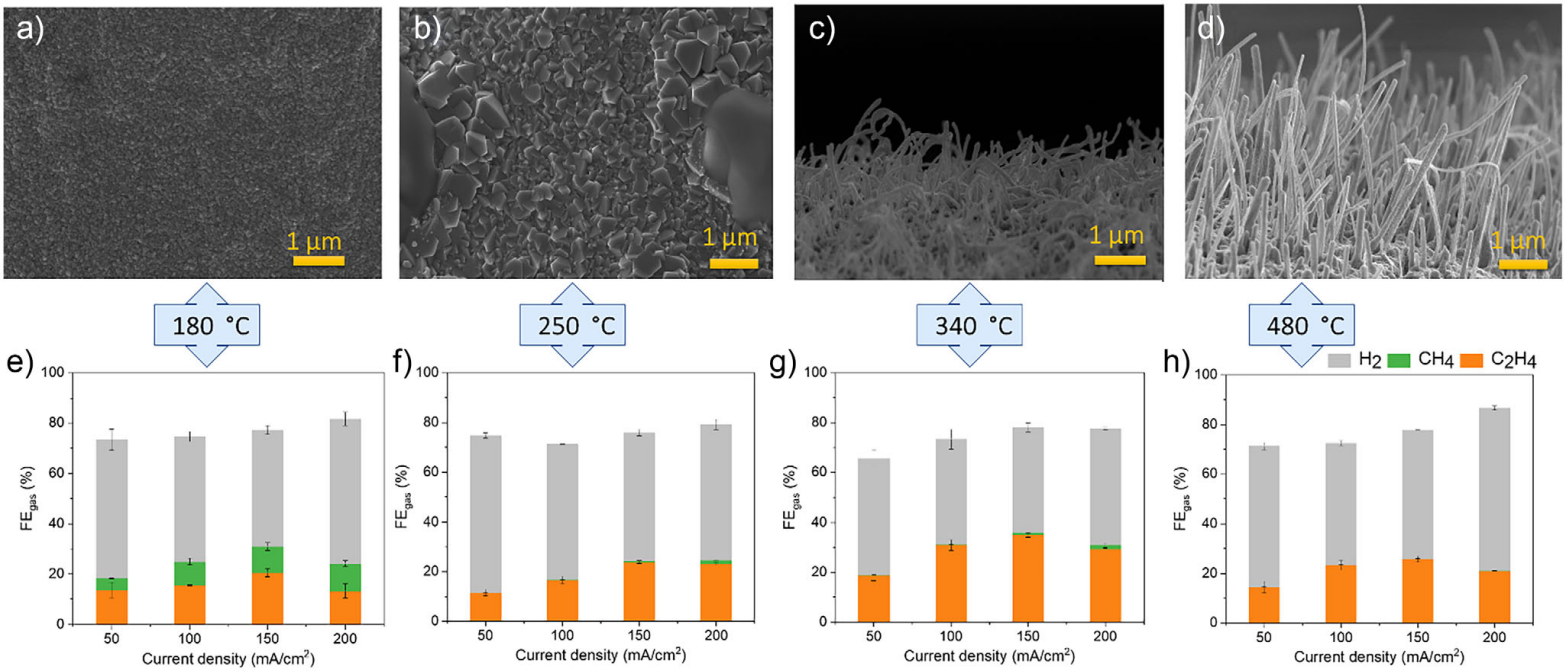
SEM images of electrochemically reduced Cu mesh catalysts, preliminarily heated at; a) 180 °C, b) 250 °C, c) 340 °C, d) 480 °C, and their corresponding electrochemical performance of bicarbonate reduction to C_2_H_4_ at various current densities e)–h). All heat treatments were performed under atmosphere conditions for 3 h.

To this end, we varied the HT temperature between 180 and 480 °C each for three hours. Cu samples were subsequently reduced via cyclic voltammetry (CV) within a potential range of −0.5 to −2 V (versus Ag/AgCl), employing a scan rate of 50 mV s^−1^ over 10 cycles in a three‐electrode H‐cell system. A representative CV curve is shown in Figure S4 for Cu treated at 340 °C for 3 h under ambient condition. As can be seen the current response decreases with increasing cyclic number (the first three cycles are shown in blue and the final three cycles in red), which is attributed to the reduction of Cu oxides, and activation of the surface. This was performed after HT at each temperature. Figure 3a‐d provides clear evidence of surface morphology variations under different HT conditions. At 180°°C, minimal agglomeration is observed (Figures 3a and S5c,d) compared to the untreated Cu mesh surface (Figure S5(a,b)) and the unreduced Cu mesh after HT (Figure S5(e,f)). However, as the temperature increases, the formation of a nano‐needle morphology becomes evident (Figure 3b‐d). Notably, at 480 °C, the surface is covered with sparser and thicker nano‐needles (Figures 3d and S6(a–c)). Electrochemical bicarbonate conversion tests showed that the sample treated at 180 °C for 3 h exhibited a maximum ∼12% FE for CH_4_ and ∼19% FE for C_2_H_4_ at current density of 150 mA cm^−2^ (Figure 3e), likely due to partial oxidation of the Cu surface. Under the HT condition of 250 °C, a maximum C_2_H_4_ FE of 24% was achieved at 150 mA cm^−2^ current density (Figure 3f). SEM images of the sample treated at 340 °C revealed a needle‐like Cu morphology with a high density of surface needles (Figures 3c and S6(d–f)). This sample achieved a maximum FE toward C_2_H_4_ of 36% at 150 mA cm^−2^ (Figure 3g). When the temperature was further increased to 480 °C, the Cu mesh was fully covered with thicker and sparser needles (Figures 3d and S6(a–c)). Higher HT temperature resulted in a drop of C_2_H_4_ FE to ∼25% at 150 mA cm^−2^, suggesting that the catalyst had surpassed its optimal HT condition.

Further characterization using X‐ray diffraction (XRD) demonstrated a mix of Cu_2_O and CuO phases in the crystalline structure of the Cu sample after HT at both 340 °C and 480 °C (Figure S7a). In contrast, the untreated Cu mesh exhibited only metallic Cu (Figure S7b), Cu treated at 250 °C showed the formation of only the CuO phase (Figure S7b). Although these oxides are reduced to metallic Cu during electrochemical reduction, oxide‐derived Cu catalysts are known to promote alternative reaction pathways and facilitate the involvement of key intermediates in eCO_2_RR. It has been shown that the local pH can increase due to the surface roughening following the reduction of Cu oxides as well as due to the specific orientation of the reduced Cu grain boundaries. All these effects contribute to enhanced FE toward C_2_H_4_.^[^ ^32^, ^33^ ^]^ Moreover, oxide‐derived Cu catalysts have shown to specifically absorb and stabilize the CO_2_^−^ radicals and leading to the formation of reaction intermediates such as *COOH and *CO. These intermediates can subsequently undergo C–C coupling to produce C_2_H_4_ and other C_2+_ products.^[^ ^34^ ^]^

Significantly, SEM images of Cu samples before surface reduction via CV at different temperatures revealed some changes in the surface morphology of the Cu samples, likely due to surface reconstruction, a well‐documented occurrence in Cu materials (Figure S8(a–h)).

Further analysis of the CH_4_/C_2_H_4_ ratio demonstrated that higher HT temperatures yield the lowest ratio (Figure S9). As we varied the HT temperatures, we achieved a maximum C_2_H_4_ FE of 34% at 150 mA cm^−2^ with a CH_4_/C_2_H_4_ ratio of 0.038. Achieving high C_2_H_4_ FE requires both a sufficiently alkaline local pH and adequate CO_2_ availability.^[^ ^35^, ^36^ ^]^

**Figure 4:**
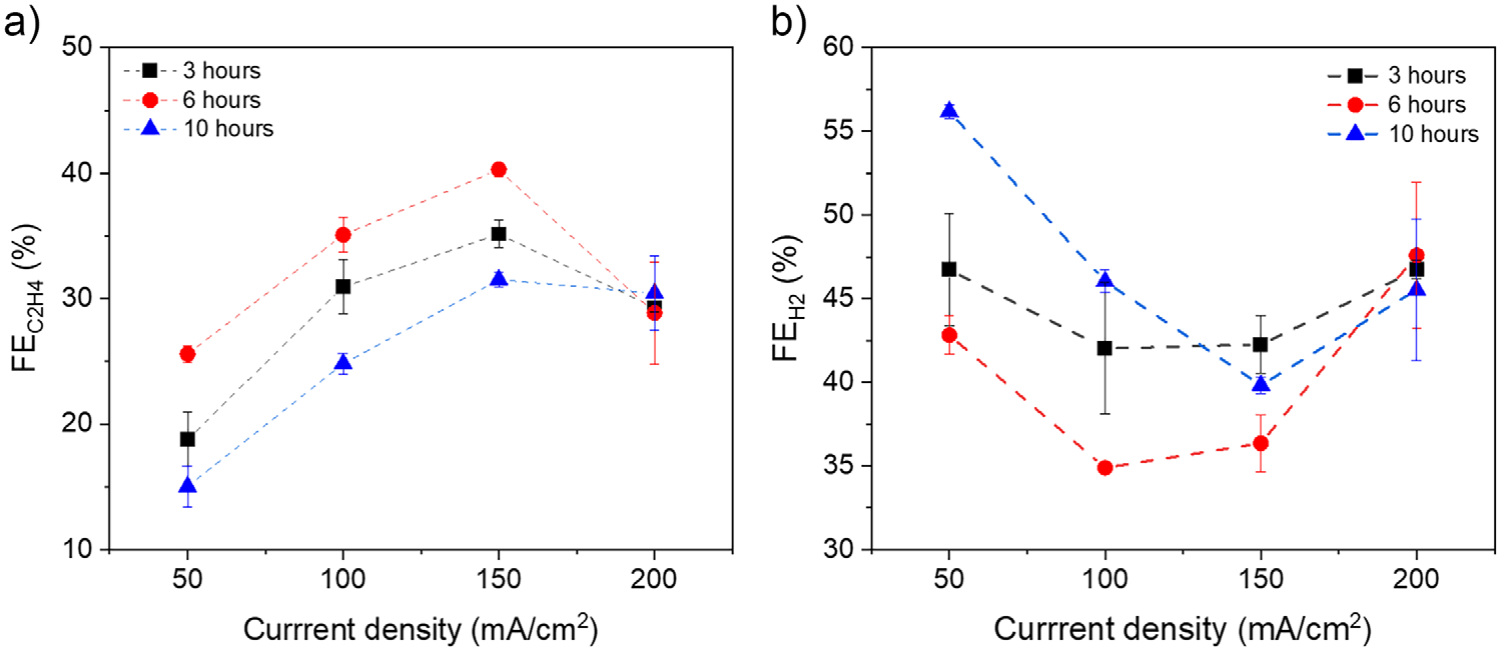
Effect of heat treatment time at 340 °C on the gas FE at different current densities, a) FE of the C_2_H_4_ production and b) the FE of H_2_ production (Cu mesh with 200*200 PPI size was used for this experiments).

Varying the HT temperature revealed that 340 °C represents an optimal condition for C_2_H_4_ production. To further optimize HT, three different HT times were considered including 3, 6, and 10 h. As can be seen from results in Figure 4, at 6 h we obtained a maximum FE of ∼39% for C_2_H_4_ at 150 mA cm^−2^ with lower H_2_ FE of ∼35%, whereas the other conditions resulted in lower C_2_H_4_ FE and higher H_2_ FE (Figure 4a,b). This difference could be due to the variations in oxide layer thickness on the Cu mesh surface, which varies among different HT times. However, the FE for methane remained below 2% for all conditions and current densities (Figure S10).

### Effect of Catholyte Composition

The presence of various cations and anions has a significant effect on CO_2_ conversion.^[^ ^37^ ^]^ In eCO_2_RR, both protons and alkali cations electromigrate to the catalyst surface and significantly modify the microenvironment of the catalyst surface (schematic representation in Figure S11a). Using large cations like K^+^ can hinder H^+^ diffusion toward the electrode surface. Due to the shielding effect of K^+^ in solution, H^+^ diffusion to the catalyst surface is limited. However, CO_2_ can easily diffuse to the electrode surface due to charge neutrality (Figure S11a). As a result, the presence of K^+^ will help to create large CO_2_ surface coverage and subsequently promote CO_2_ reduction. Also, anions (such as Cl^−^) can regulate the FE by affecting adsorbed ^*^CO intermediates on the catalyst surface. To further study this effect, we tested different concentrations of KHCO_3_ (Figure S11b) and the effect of K^+^ and Cl^−^ presence in the solution to show their effects. We observed that 0.1 M KHCO_3_ mixed with 0.9 M KCl has the highest FE and activity toward bicarbonate conversion into C_2_H_4_ (max C_2_H_4_ FE of 36%) (Figure S11(b,c)). Although this catholyte achieved high C_2_H_4_ FE, further investigation revealed that the performance was not solely dependent on KCl, as similar results were obtained without it (Figure S11b). Additionally, the lower KHCO_3_ concentration improves CO_2_ dissolution, enhancing CO_2_ availability at the catalyst surface and contributing to the greater selectivity toward C_2+_ products. Although higher KHCO_3_ concentrations (e.g., 0.5 M) resulted in lower C_2_H_4_ FE, the optimized Cu mesh catalyst and favourable mass transport conditions played crucial roles in achieving the observed performance.^[^ ^34^ ^]^ Additionally, a catholyte with lower HCO_3_^−^ concentration reduces buffering capacity, resulting in a higher local pH on the catalyst surface during electrochemical reduction; accordingly, higher C_2_H_4_ FE was observed with lower KHCO_3_ concentration (Figure S11a). To further investigate the effect of electrolyte composition on C_2_H_4_ production, we tested KBr and KI as supporting electrolytes in our system. Our experimental results indicated that the inclusion of KBr and KI instead of KCl did not result in a significant difference in C_2_H_4_ FE compared to our standard electrolyte composition (0.1 M KHCO_3_ + 0.9 M KCl) (Figure S11d). This suggests that, within our experimental conditions, the presence of these halide anions does not affect the production of C_2_H_4_ significantly.

### Effect of Cu Mesh Pore Size

After optimizing the catholyte composition and HT conditions, we investigated the effect of Cu mesh pore size on the direct electroreduction of bicarbonate to C_2_H_4_. We tested Cu meshes with various pore sizes, including 60*60, 80*80, 100*100, and 200*200 wires per linear inch. As the meshes become finer, the C_2_H_4_ FE performance generally improves. The 60*60 mesh showed a maximum C_2_H_4_ FE of ∼15% at 150 mA cm^−2^, while the 200*200 mesh had a C_2_H_4_ FE of ∼33% at 150 mA cm^−2^ and the lowest H_2_ FE of 42% (Figure 4a,b). The FE for CH_4_ is also shown in Figure S12, and as can be seen, the methane FE for all samples is less than 5% at all current densities. We have identified two potential effects associated with changing mesh sizes. First, finer meshes are thinner, resulting in shorter effective catalyst domains. Modelling predicts that only very thin zones (approx. 1 µm) exhibit active CO_2_ reduction, while HER dominates in the rest of the domain. Thus, shortening the domain would presumably improve C_2_ FE.

After optimizing the parameters for direct bicarbonate electroreduction to C_2_ products on HT Cu mesh, we conducted further evaluations on the liquid products produced using Cu mesh treated at 340 °C for 6 h at different current densities (the optimized condition). The analysis performed using 1H NMR spectroscopy revealed significant findings. A representative NMR profile is shown in Figure S13a. At a current density of 150 mA cm^−2^, we achieved a maximum ethanol FE of 13.5% (Figure S13b). Additionally, our investigation showed a maximum formate FE of approximately 5.6% at 100 mA cm^−2^ and propanol FE of 3.31% at the same current density (Figure S13). Combining the C_2+_ products from the liquid phase, our results indicate an overall C_2+_ liquid production of nearly 17%. Notably, C_2_H_4_ production accounted for approximately 39% of the total C_2+_, resulting in an impressive C_2+_ products FE of over 55% under optimal conditions. The SEM images of the Cu mesh sample treated at 340 for 6 h before and after reduction are shown in Figure S13(c–f), which are consistent with other observations for different HT conditions.

### Conversion Efficiency

We measured the total *i‐CO_2_* generated from the reaction between H^+^ and HCO_3_^−^ (Equation 1) for the heated Cu mesh to determine the correlation between *i‐CO_2_* generation and its utilization efficiency (Figure S14(a,b)). The *i‐CO_2_* was quantified by measuring the combined concentrations of C_2_H_4_, CH_4_, and unreacted CO_2_ leaving the electrolyzer, determined by GC measurement. We obtained an *i‐CO_2_* generation concentration of nearly 3 mM (Figure S14a). The conversion efficiency was calculated using Equation 5 of the Supporting Information. Figure S14b shows a conversion efficiency of over 90% at all current densities, from 50 to 200 mA cm^−2^, with somewhat reduced efficiencies at higher current densities. Achieving high CO_2_ utilization efficiency and ensuring that the gas product stream is free of CO_2_ brings substantial economic advantages as it minimizes the need for costly separation processes following eCO_2_R.^[^ ^38^, ^39^ ^]^

**Figure 5:**
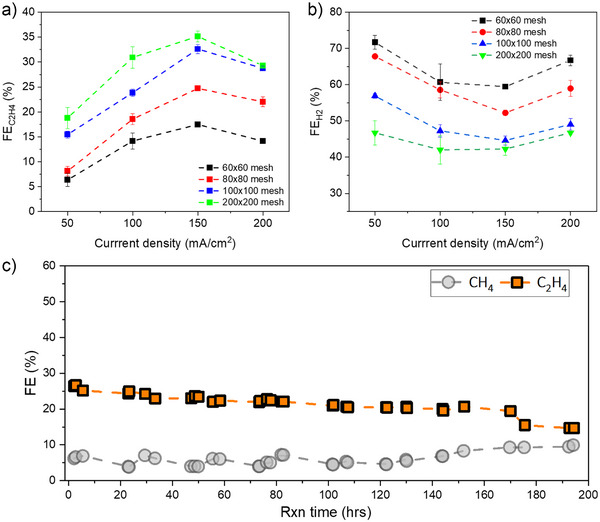
Effect of Cu mesh pore size on FE for C_2_H_4_ at different current densities, a) FE of the C_2_H_4_ production and b) the FE of H_2_ production, and c) long‐term stability test within simulated carbon capture condition from 10% CO_2_ containing gas stream (10% CO_2_ + 90% N_2_) under 5 min “On” and 2.5 min “Off” segments. Note that all samples are HT at 340 °C for 3 h.

To evaluate the stability of the catalyst, we conducted bicarbonate conversion at a constant current density of 150 mA cm^−2^ and tracked the gas products over operation time. The system demonstrated a high FE for C_2_H_4_ between 35 to 40% for around 4 hours (Figure S15a). However, hydrogen FE increased over time (from 36% to 58%, Figure S15b), and the FE for CH_4_ slightly increased from 0.5% to 3.36% over the operation time (Figure S15b). Inspired by CO_2_ capture from flue gas and previous studies on the effect of pulse electrolysis on Cu catalyst stability,^[^ ^40^, ^41^ ^]^ we conducted a stability test under simulated carbon capture conditions. In this experiment, we purged the bicarbonate capture solution with a gas stream mixture of 10% CO_2_ and 90% N_2_ and applied pulse electrolysis. The electrolysis consisted of 300s “On” segments at a current density of 100 mA cm^−2^, followed by 200s “Off” segments. Under these conditions, our catalyst operated for over 160 h with an C_2_H_4_ FE of 20–25% (Figure 5c). After almost 160 h the CH_4_ and H_2_ FE began to increase (Figures 5c and S15c). We attribute this long‐term stability to the chemical and homogeneous oxidation of Cu atoms on the catalyst's outermost surface during the “Off” periods, and CO_2_ diffusion to the catalyst surface during the “Off” segments, which suppresses HER. We characterized the Cu mesh surface using SEM and XRD after 195 h of operation (Figure S16). The SEM images show surface reconstruction of the Cu (Figure S16 a–c), and XRD analysis revealed mostly metallic peaks with an intensified CuO peak (Figure S16d).

## Conclusion

In this work, we show that tuning Cu mesh porosity and thickness enables the selective electrosynthesis C_2_H_4_ from a reactive bicarbonate solution with less than 10% volume detected CO_2_ gas in the outlet gas stream (all experiments were conducted under N_2_ purging). This demonstration was achieved through the systematic optimization of catalyst surface oxidization, porosity, thickness, and catholyte composition. Our results show that a cathode with a large pore size in the micron scale increases the rate of HCO_3_^−^ transport, enabling higher *i‐CO_2_* utilization and resulting in a high C_2_H_4_ FE of 39% and total C_2+_ product FE of over 55% at 150 mA cm^−2^. More importantly under simulated CO_2_ capture from flue gas (10% CO_2_ + 90% N_2_) and on/off electrolysis, the catalyst was stable for more than 160 h at a current density of 100 mA cm^−2^. The bicarbonate electrolyzer system generates a gas stream with high (>90%) CO_2_ utilization that is undiluted by CO_2_, thereby potentially reducing downstream regeneration/separation costs, as supported by existing literature.^[^ ^12^ ^]^

## Supporting Information

Characterization and methods, electrochemical data, XRD patterns, and SEM images. Supporting Information is available free of charge.

## Author Contributions

The manuscript was written through contributions of all authors. All authors have given approval to the final version of the manuscript.

## Conflict of Interests

CERT is a company commercializing electrochemical CO_2_ utilization technology.

## Supporting information



Supporting Information

## Data Availability

The data that support the findings of this study are available from the corresponding author upon reasonable request.
