# Supplementary material for: Selective Electrochemical Production of Ethylene from Bicarbonate Solution
Source: Angew Chem Int Ed Engl. 2025 Jul 17;64(35):e202509975. doi: 10.1002/anie.202509975 (PMC12377452; doi:10.1002/anie.202509975)
Supplement: Supplementary file 1 — Supporting Information [file ANIE-64-e202509975-s001.docx]

**Supplementary information**

**Selective Electrochemical Production of Ethylene from Bicarbonate Solution**

Behnam Nourmohammadi Khiarak^1^, Gelson T. S. T. da Silva^1,2^, Jackson Crane^3^, Colin P. O’Brien^4^, Michael R. Pepe^4^, Christine M. Gabardo^4^, Viktoria Golovanova^5^, F. Pelayo Garcia de Arquer^5^, and Cao-Thang Dinh^1,*^

^1^Department of Chemical Engineering, Queen’s University, Kingston, Ontario, Canada K7P 3N6

^2^Interdisciplinary Laboratory of Electrochemistry and Ceramics, Department of Chemistry, Federal University of Sao Carlos, São Carlos, SP, 13565-905, Brazil

^3^Department of Mechanical and Materials Engineering, Queen’s University, Kingston, Ontario, Canada K7P 3N6

^4^CERT System Inc., Toronto, ON, M6N 2J1, Canada

^5^ICFO-Institute de Ciencies Fotoniques, The Barcelona Institute of Science and Technology, Barcelona, 08860, Spain

*Corresponding author. Email address: [caothang.dinh@queensu.ca](mailto:caothang.dinh@queensu.ca)

**Method**

**Materials**

The Cu meshes in various sizes were purchased from Amazon. These meshes have a purity of 99.9% Cu, are polished, and are manufactured by TUSEA under the Generic brand. Acetic acid (high purity, 99%), ethanol, Millipore water, deuterium oxide (D_2_O), potassium bicarbonate (KHCO_3_, Aldrich, 99%) and potassium chloride (KCl, Aldrich, 99%) were used to conduct the experimental works.

**Electrochemical bicarbonate reduction**

Electrochemical measurements were performed in a two-electrode MEA flow cell. A potentiostat (Autolab PGSTAT204) with a current booster (Metrohm Autolab, 10A) was used for all experiments. Nickel foam (1.5 mm thickness; 80-100 pores per inch (ppi), MTI Corp.) and copper mesh (60-200 ppi) were used as the anode and cathode, respectively. The exposed size of the cathode and anode electrode was 1 cm x 2 cm (a geometric area of 2 cm^2^ was used for all current density calculations). The anolyte was 1 M KOH and the catholyte was mixture of KCl and KHCO_3_ saturated with N_2_ gas. A bipolar membrane (Fumasep) was used to separate anode and cathode. The copper mesh was configured to be in direct contact with the membrane. A polypropylene (PP) mesh-type spacer was used to avoid direct contact between the Cu mesh and the flow channel. Two peristaltic pumps were used to deliver the anolyte and the catholyte to their respective compartments. N_2_ was continuously purged into the catholyte throughout the experimental process at a flow rate of 45 standard cubic centimeters per minute (sccm).

To conduct cyclic voltammetry, a H-cell was used. The anode was made of Pt mesh and cathode was our working electrode and the reference electrode was Ag/AgCl (3M KCl). And the anode and cathode were separated with a BPM. The anolyte was 1 M KOH and catholyte was 0.1M KHCO_3_ + 0.9 M KCl with a volume of 50 mL for both.

The gaseous products were gathered from the cathode electrolyte reservoir and analyzed for quantification using online gas chromatography (GC) employing the PerkinElmer Clarus 590 instrument. The GC system utilized in the study was equipped with a thermal conductivity detector (TCD) and a flame ionization detector (FID). It was directly connected to the outlet gas stream of the cell, enabling continuous online analysis. Additionally, aliquots of the liquid products were subjected to analysis using nuclear magnetic resonance spectroscopy (NMR). The 1H NMR spectra of the freshly collected liquid products were obtained using an Auto-400 ultrashield Bruker Instrument. The spectrometer operated at a specified frequency in megahertz (MHz) at a temperature of 25°C in D_2_O, employing water suppression mode. Dimethyl sulfoxide (DMSO) served as the internal standard for calibration.

To calculate the FE of gas products, the following formula (Eq. 3) was used:

FE_i_ = $\frac{n_{i}*F*x_{i}*F_{m}}{I}*100\%$

In above equation, FE_i_ represents the FE of product i, n_i_ is the number of electrons (for i = C_2_H_4_, n_i_ is 12, for CH_4­_ it is 8) transferred to produce gas product i, F is Faraday’s constant (96485 C mol^-1^), F_m_ is the molar flow rate of the gas outlet stream in mol.s^-1^, x_i_ is the molar fraction of the gas product i in the gas outlet stream during ECR. I isthe total applied current in Ampere (A).

The FE of bicarbonate electroreduction to liquid products distribution were calculated as follows (Eq. 4):

FE_i_ (%) = $\frac{n * F * \eta}{J*t}$ (Eq. 3)

where n is the number of electrons transferred to form liquid product i, J is the total current, η which is the number of moles of the resultant product, and t is electrolysis time.

The conversion efficiency, CE, was calculated as follows:

CO_2_ conversion efficiency = $\frac{{moles of the CO}_{2}conversion products}{moles of CO2 conversion products+moles of unreacted CO2}$ (Eq. 5)

CO_2_ conversion products that are considered here is: CH_4_, C_2_H_4_, C_2_H_5_OH, CH_3_CH_2_CH_2_OH, and CH_3_COOH.

**Characterisations**

The surface morphology of the prepared samples was analyzed through scanning electron microscopy (SEM, Thermo Fisher, Quanta 250) at Queen’s University department of Geological Sciences and Geological Engineering. The SEM was operated at 15 KV. XRD analysis was conducted using a Thermo Scientific K-Alpha spectrophotometer with a monochromated Co Kα X-ray radiation source. For analysing the obtained XRD spectra, we have converted the Co radiation source to Cu radiation source to match the data with literature.

**Multiphysics model**

A one-dimensional multiphysics model was used to predict key species concentrations and electrocatalytic rates. The modelled domain includes the catalyst layer and the cation exchange layer (CEL) portion of the bipolar membrane. Four catalyst layer widths were considered, 75 µm, 250 µm, 500 µm, and 1500 µm. In all cases, the CEL width is 75 µm. The transport and buffer reaction dynamics of aqueous HCO_3_^–^, K^+^, CO_3_^2–^, OH^–^, H^+^, and CO_2_ are considered in the model. Dilute solution theory is used to describe the transport with the Nernst-Planck equation. In this domain, convection is assumed to be negligible in the model direction (convection in the other dimensions are accounted for in the boundary conditions). Electroneutrality is imposed. The effective diffusion coefficient in the porous catalyst domain is determined with the Bruggeman correction, with a porosity of 0.99. A gas fraction of 0.2 is assumed in the catalyst layer, matching previous studies [1, 2]. The current distribution is determined with Ohm’s law and the electrochemical kinetics are described by the concentration-dependent Butler-Volmer equation [3]. To simplify the modelling, only the hydrogen evolution reaction and a 12-electron C_2_ reaction (corresponding to ethylene and ethanol evolution) are considered, matching the major products found in experiments. The electrochemical rates are fit to the experimental performance of the copper mesh catalyst. The Donnan equilibrium boundary condition was used to describe the charge discontinuity between the copper catalyst and the CEL. The CEL has a prescribed fixed space charge density of -1.75 C m^-3^ [4]. The water hydration (mol H_2_O per mol SO_3_^–^) in the CEL was determined to be 6, which defines CEL diffusivity [5]. Henry’s law was used to model CO_2_ phase transfer from liquid to vapor phase. It is assumed that any CO_2_ bubbled away will not re-dissolve into solution due to the fast timescales associated with bubble advection.[2, 6]The domain considered is the catalyst layer and the CEL portion of the BPM. The left boundary condition of the simulation (as in Figure 2a) corresponds to the interface between the flow plate and the catalyst layer. A mass flux boundary condition is imposed at this interface with a constant Sherwood number, corresponding to laminar flow. An assumed Sherwood number of 36.6 was used to account for unsteady convection increasing catholyte flux [1]. The bulk catholyte concentration is 0.1 M KHCO_3_, with the full equilibrium constituent concentrations listed in Table S2. We assume that the 0.9 M KCl used in experiments only affects the environment local to the catalyst, and therefore is accounted for in electrocatalytic rate fitting. The solid-phase electric potential on the flow-plate interface is set to the half-cell potential (versus SHE), varied from 0 V to -2 V. The boundary condition on the right side of the domain corresponds to the interface between the CEL and the anion exchange layer, within the BPM. A boundary flux of protons proportional to the integrated current catalyst domain density times a transference number of 0.75 is used [1, 2]. A transference number of 0.75 is used. The net ionic current is from K^+^ across the membrane. All other species have a no-flux condition at this interface. The electrolyte potential at this boundary is set to 0 V.

COMSOL Multiphysics version 6.1 using the PARDISO solvetabr was used to solve the model. The 1D domain is discretized into elements of maximum size 0.25 µm with elements of 0.02 µm near boundaries. Additional parameters are included in Table S2.


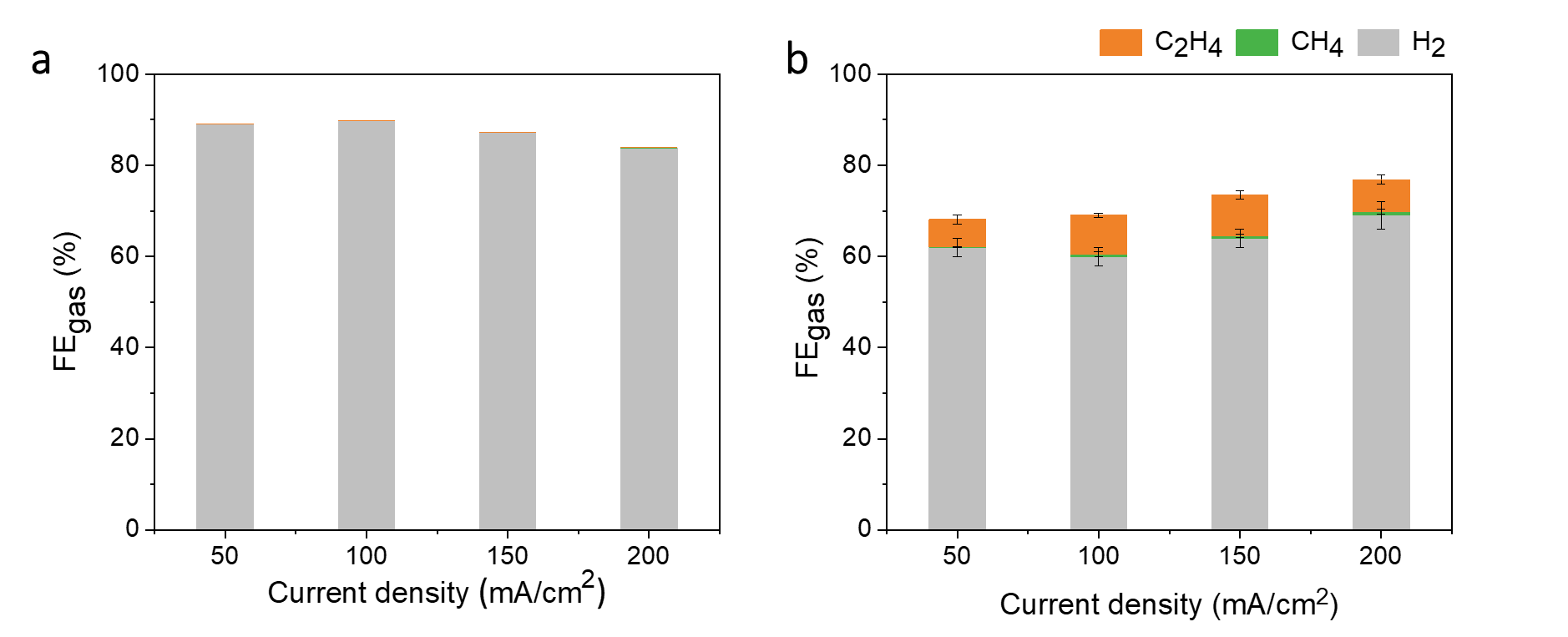


**Figure S1**: The FE for gas products on (a) untreated Cu mesh sample and (b) heat treated Cu mesh (200*200 PPI mesh size) at 340 °C for 3 hrs, the experiments were done at 1 M KHCO_3_ in MEA cell configuration.

**Table S1**: a comparison of recent reported works on bicarbonate/carbonate electroreduction to multi-carbon products.

| Catalyst | Electrolyte | Cell-Type | Product FE (%) | Current density (mA/cm^2^) | Cell Voltage (V) | CO_2_ utilization (%) | Ref. |
| --- | --- | --- | --- | --- | --- | --- | --- |
| Cu/CoPc-CNTs | 0.5 - 2 M K_2_CO_3_ | MEA/BPM | 34% C_2_H_4_ | 300 | 4.1 | 100 | [10] |
|  |  |  | 47% C_2+_ |  |  |  |  |
| Cu-Ag_0.5_ | 5 M K_2_CO_3_ | MEA/BPM | 10% C_2_H_4_ | 100 | 3.8 | 100 | [11] |
| CuSus/Ag-NafP_50_ | 3 M KHCO_3_ | MEA/BPM | 20% C_2_H_4_ | 100 | 3.65 | 4 | [12] |
|  |  |  | 41 % C_2+_ |  |  |  |  |
| OD-Cu/C | 0.1 M KHCO_3_ | MEA/BPM | 26% C_2_H_4_ | 60 | 3.4 | 80 | [13] |
|  |  |  | 48 % C_2+_ |  |  |  |  |
| Cu mesh/Heat treated | 0.1 M KHCO_3_ + 0.9 M KCl | MEA/BPM | 37% C_2_H_4_ | 150 | 4 | > 90 | **This work** |
|  |  |  | ~65% C_2+_ |  |  |  |  |


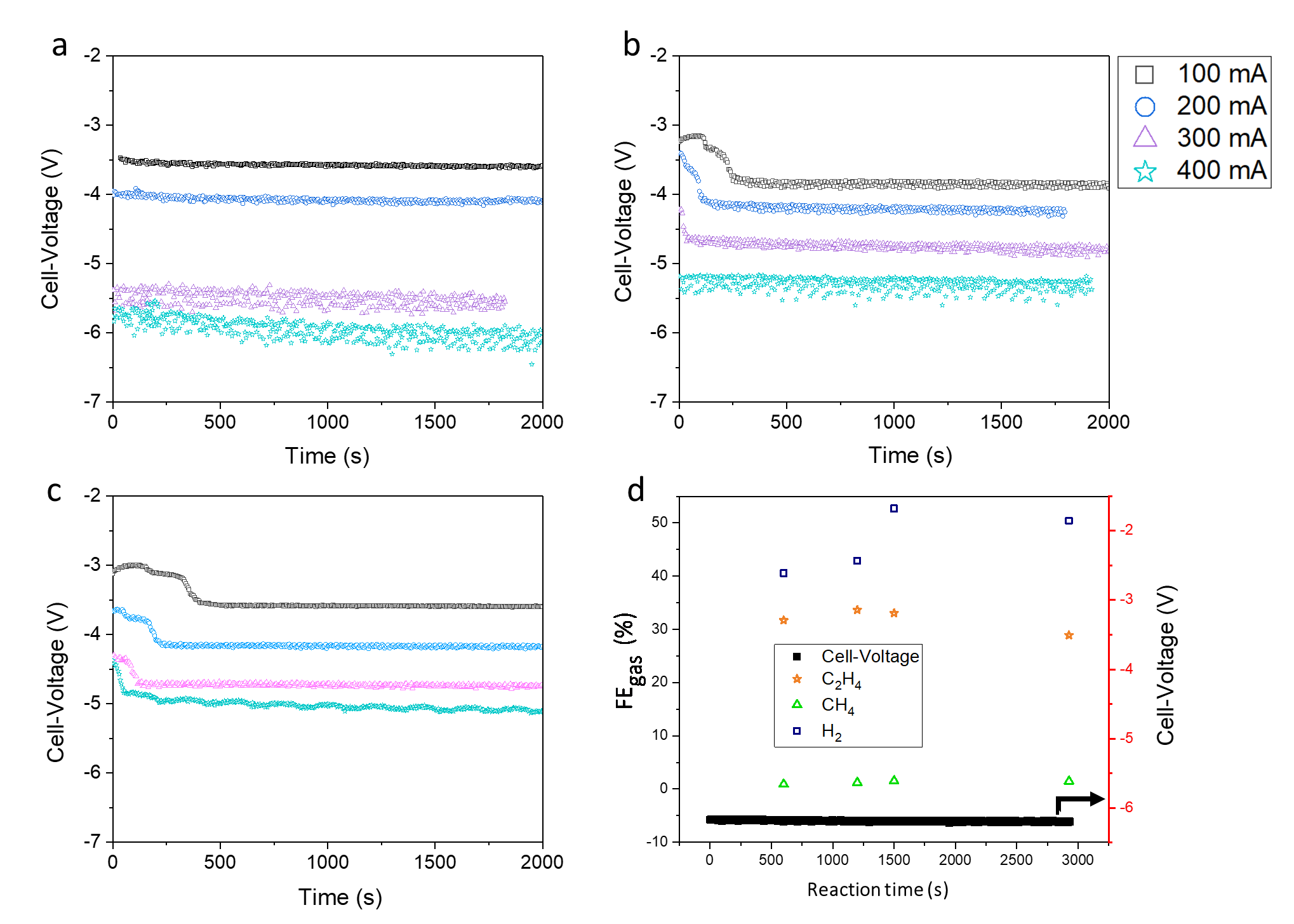


**Figure S2**: Chronopotentiometry data for different cathode configurations; (a) 1 layer Cu mesh, (b) 3 layers of Cu meshes, (c) 5 layers of Cu meshes, all samples are heat treated at 340 °C for 3 hours, and (d) the changes of FE for gas products over time of online injection to GC.

**Figure S3**: Modeled CO_2_ concentration within domain for various catalyst domain lengths.

**Figure S4**: Cyclic voltammetry (CV) curves showing the reduction of the Cu oxide surface on a Cu electrode. The potential range was scanned from -0.5 V to -1.50 V vs. Ag/AgCl at a scan rate of 50 mV/s for Cu sample HT at 340 °C. The initial 3 CV cycles are shown in blue color, and the last 3 peaks are shown in red color, as can be seen the current increment after 20 cycles stabilized and reached a constant current response.


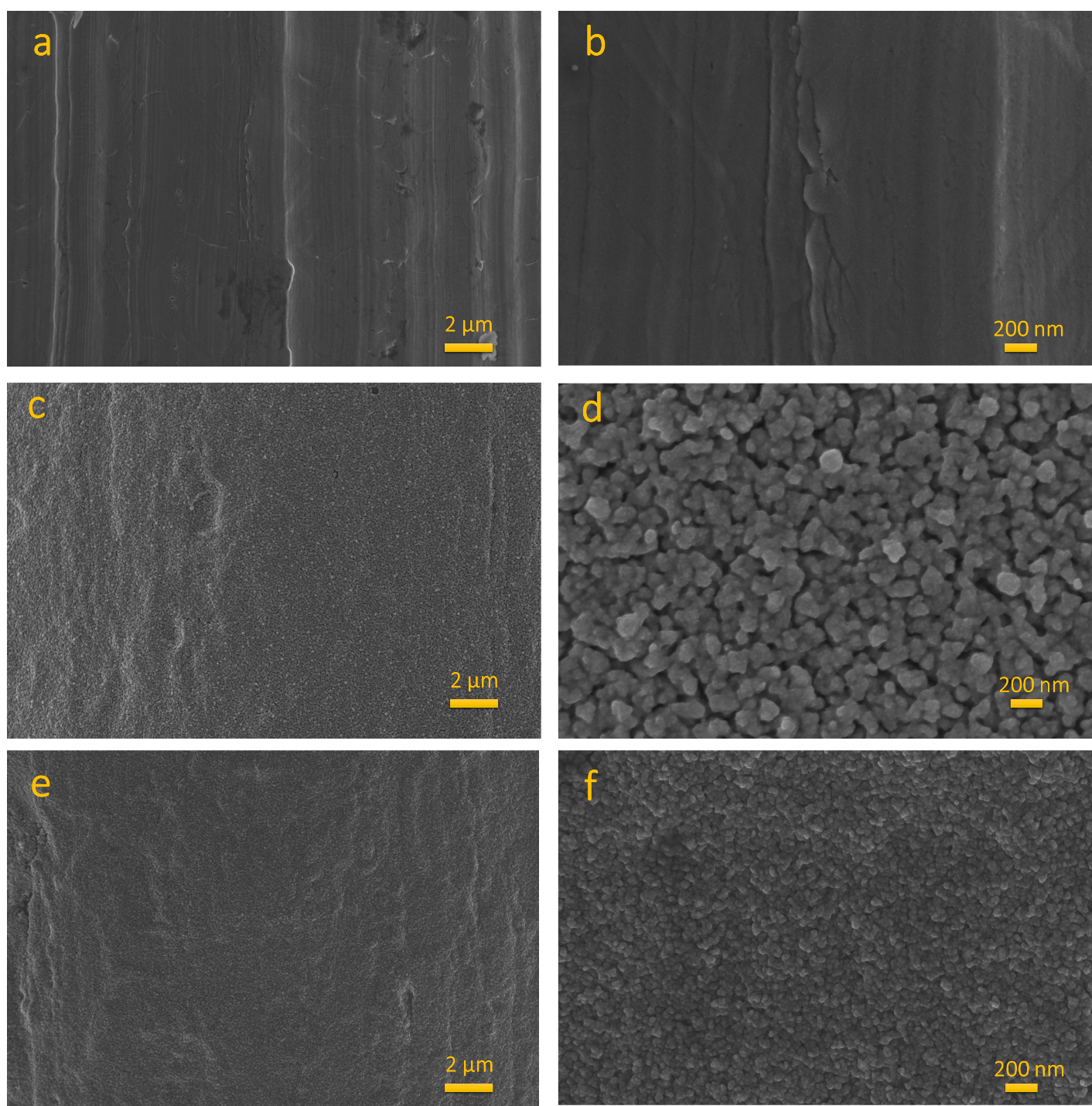


**Figure S5**: The SEM images for (a, b) untreated 2D Cu mesh surface, (c, d) treated Cu mesh surface at 180 °C for 3 hours after reducing in H-cell with CV, and (e, f) treated Cu mesh surface at 180 °C for 3 hours before surface reduction.


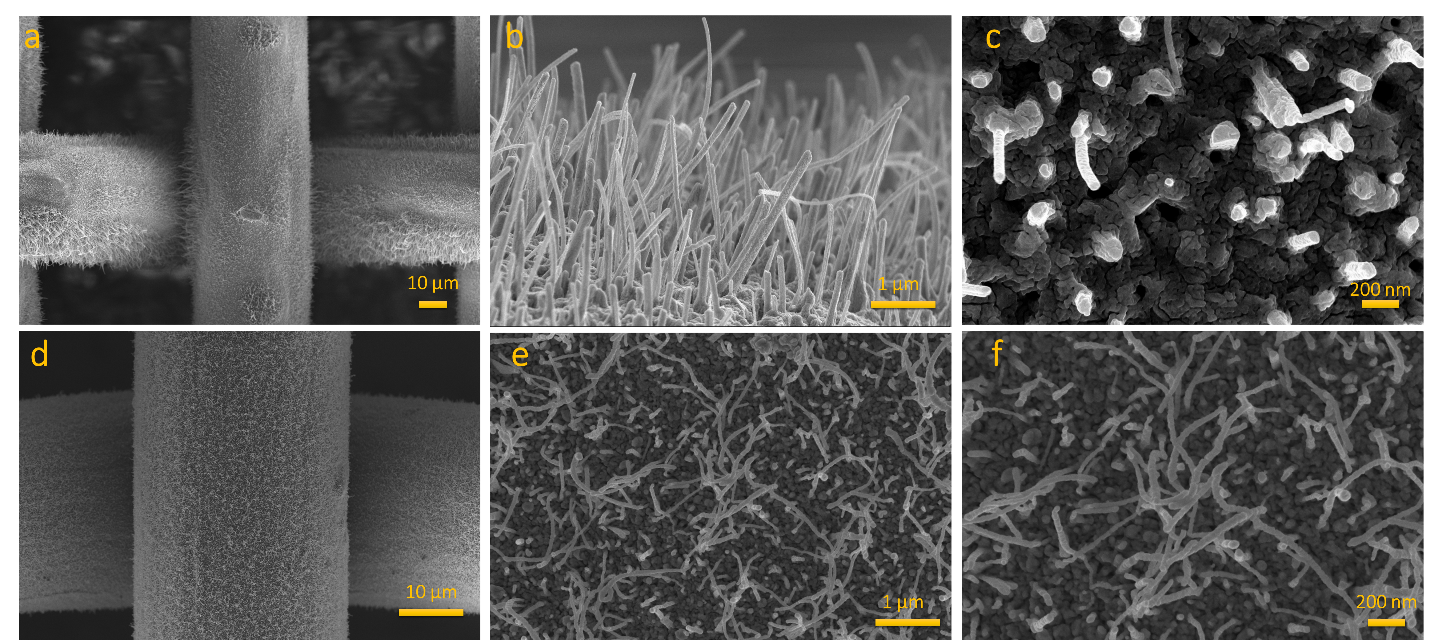


**Figure S6**: The SEM images for (a-c) Cu mesh treated at 480 °C for 3 hours at different magnifications, and (d-f) Cu mesh treated at 340 °C for 3 hours at different magnifications.


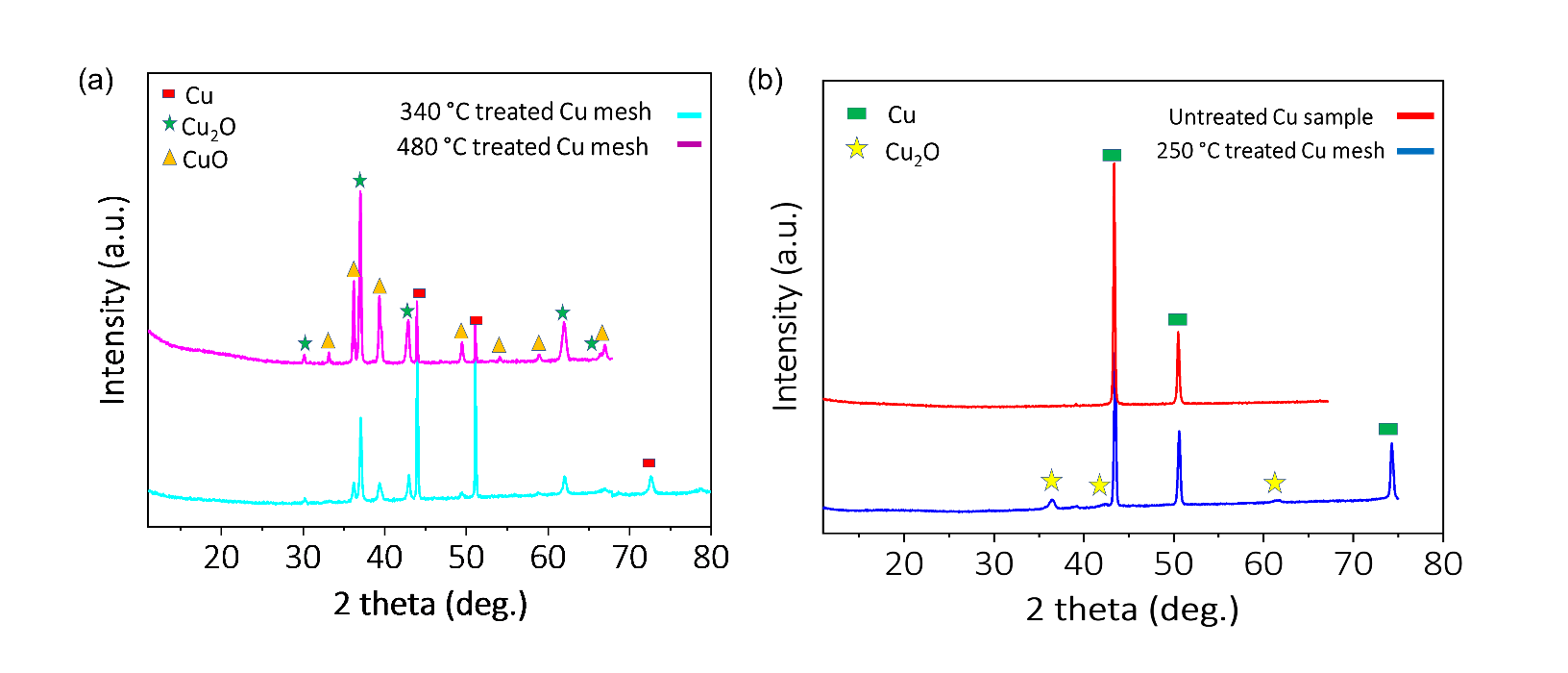


**Figure S7**: XRD profile for Cu mesh HT under (a) 340 °C and 480 °C and (b) 250 °C and untreated Cu.


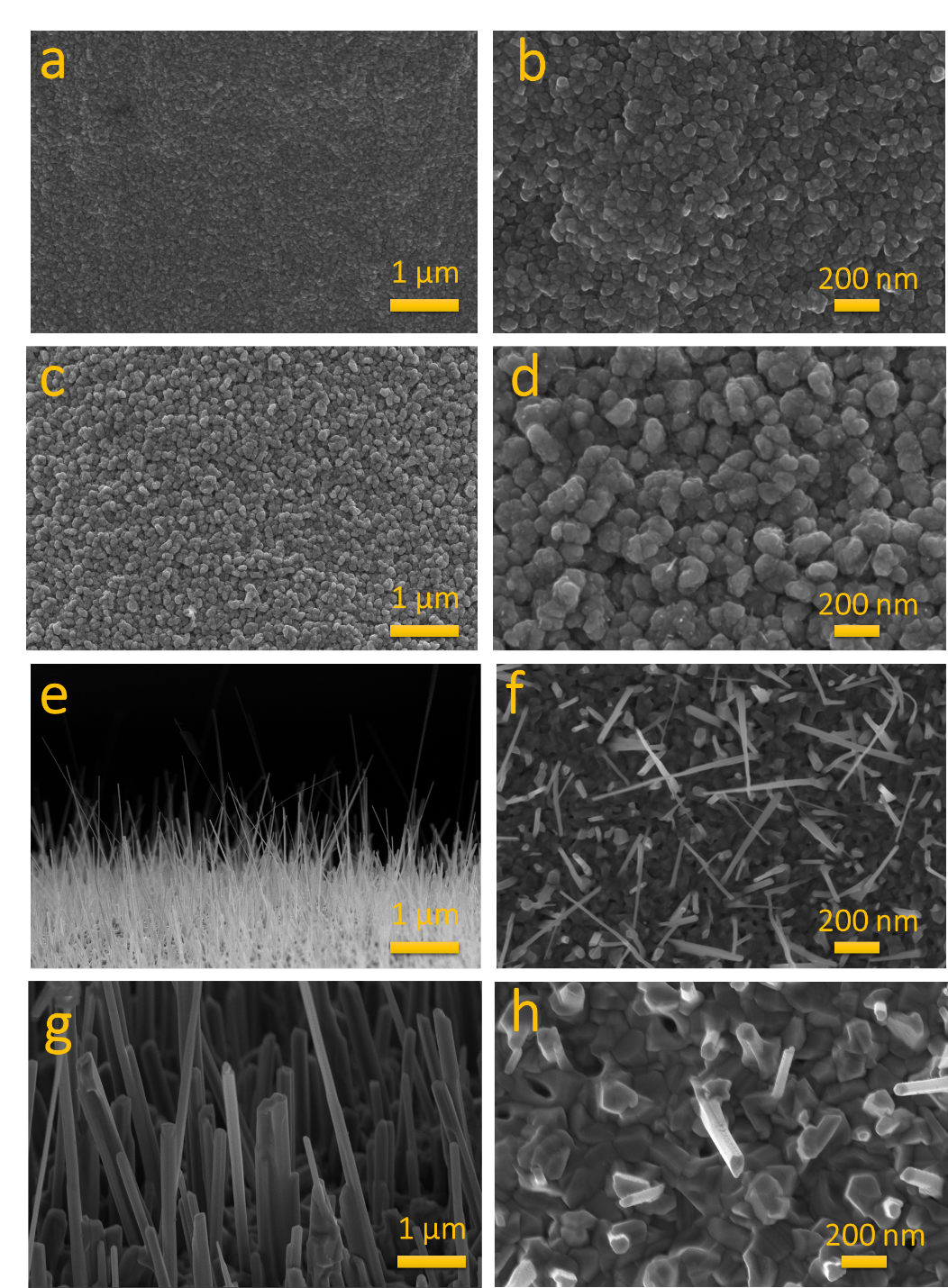


**Figure S8**: The SEM images of the Cu mesh samples before reducing under cyclic voltammetry (as-prepared samples); (a, b) Cu mesh HT at 180 °C, (c, d) Cu mesh HT at 250 °C, (e, f) Cu mesh HT at 340 °C, and (j, h) Cu mesh HT at 480 °C.

**Figure S9**: The ratio of CH_4_/C_2_H_4_ at different HT temperatures that used to screen Cu mesh samples.

**Figure S10**: The effect of heating time at 340°C heat treatment temperature at different current densities, these tests have done on Cu mesh size of 200*200.


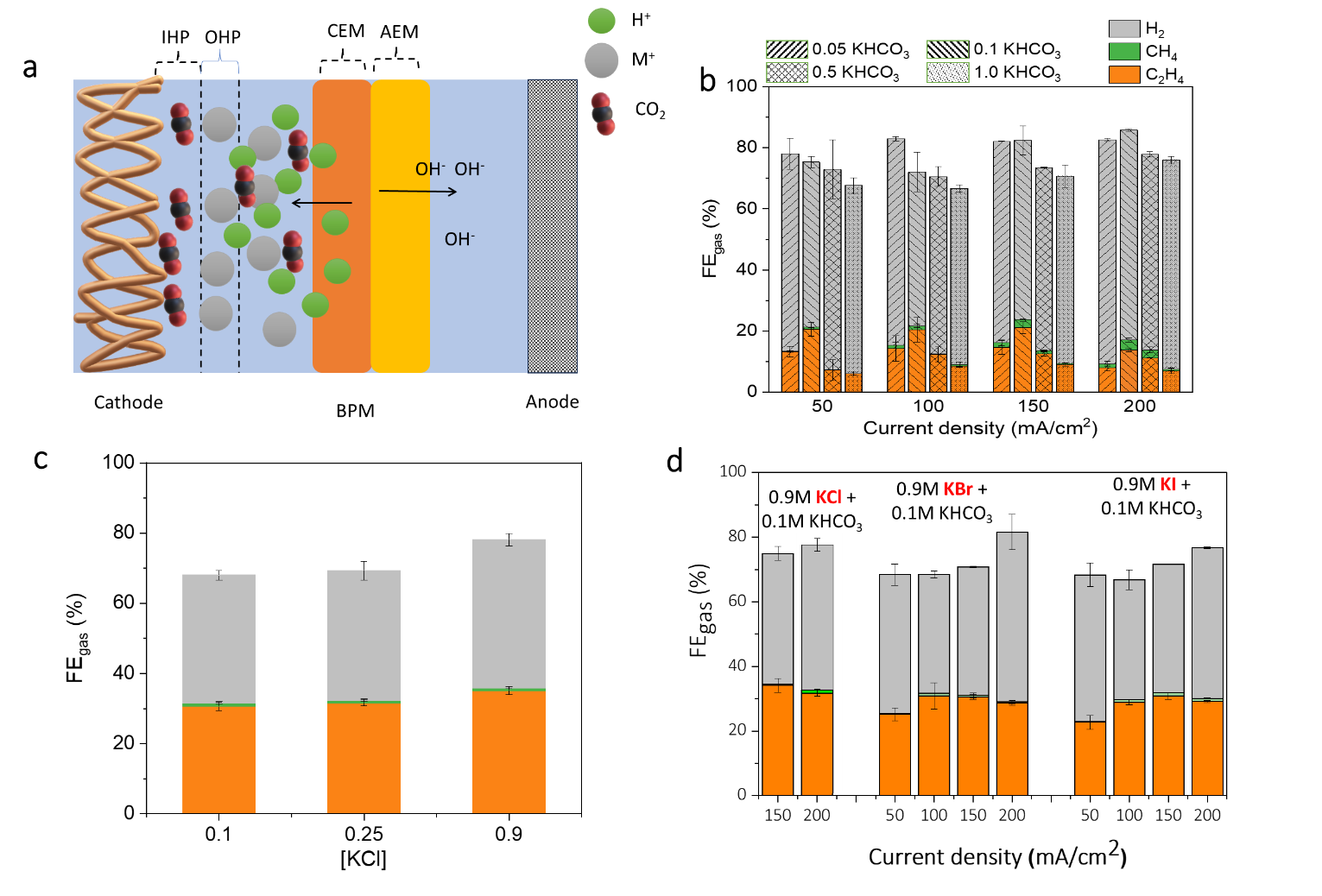


**Figure S11**: (a) A schematic representation for K^+^ cation effect on shielding H^+^ and increasing the CO_2_ availability on local surface, (b) The effect of KHCO_3_ concentration on ethylene selectivity under different applied current densities and (c) the effect of different KCl concentration in 0.1 M KHCO_3_ solution under applied current density of 150 mA/cm^2^, (d) a comparison between different anions effect on CO_2_ electroreduction effect KCl, KBr, and KI.

**Figure S12**: CH_4_ selectivity for different mesh sizes at different current densities.


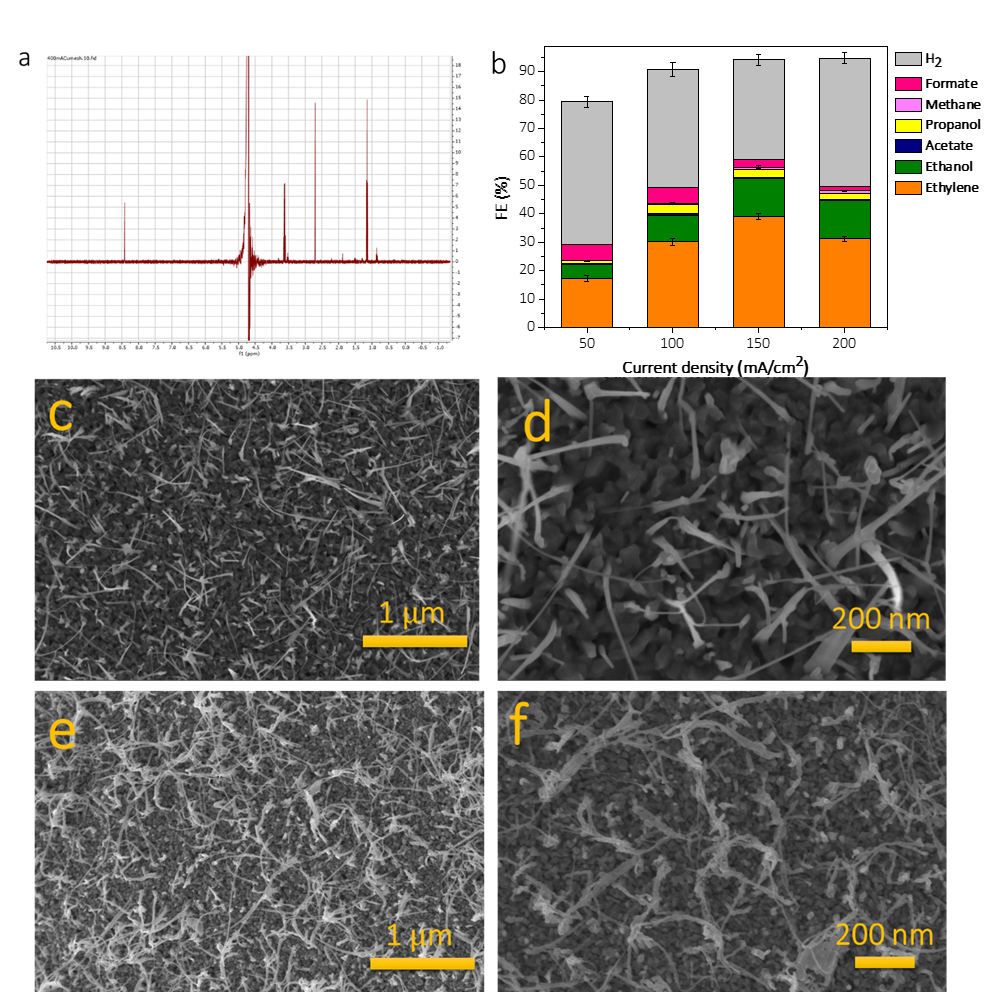


**Figure S13**: (a) a representative NMR profile demonstration for the liquid products, (b) liquid product distribution on Cu mesh sample heat treated at 340 °C for 6 hours, and the SEM images for the sample before (c, b) and after reduction (e, f).


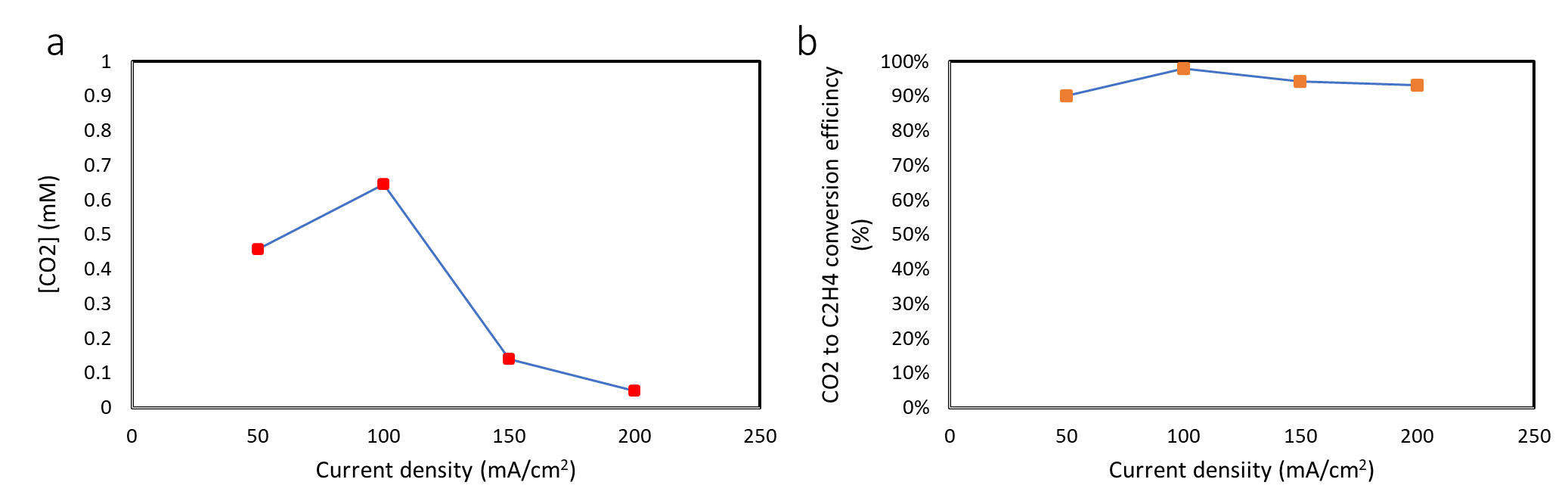


**Figure S14**: (a) the concentration of i-CO_2_ generated in the from bicarbonate system at different current densities, and (b) conversion efficiency of CO_2_ to C_2_H_4_ (%).


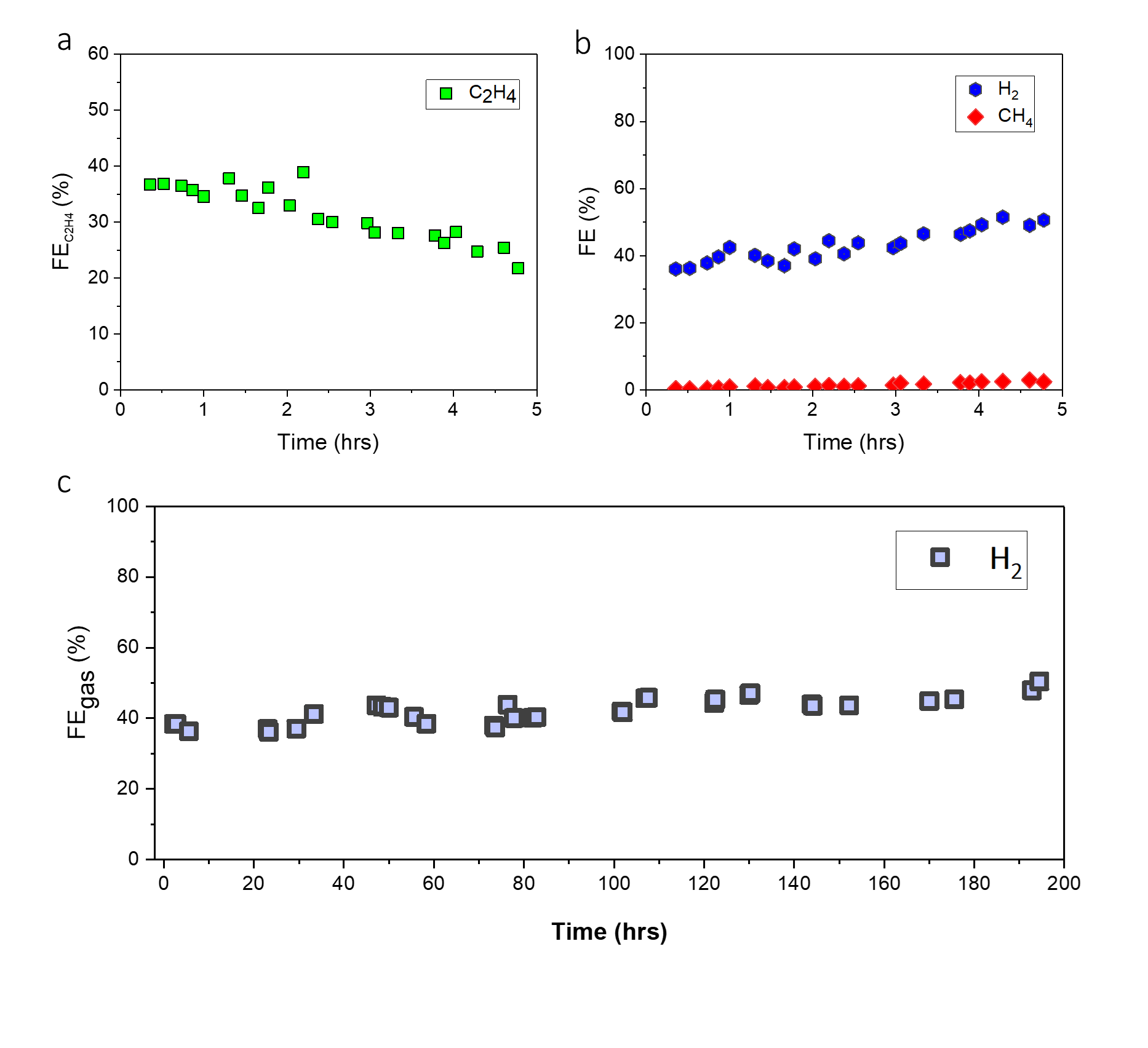


**Figure S15**: The stability result for (a) Ethylene production from direct conversion of bicarbonate solution, and (b) H_2_ and CH_4_ production selectivity over reaction time, the bicarbonate conversion was conducted under 150 mA.cm^-2^ current density in 0.1 M KHCO_3_ + 0.9 M KCl catholyte under N_2_ purging condition, and (c) the H_2_ FE under simulated carbon capture from flue gas and under segments of “On” and “Off” conditions with a current applied 100 mA/cm^2^ during on segments.


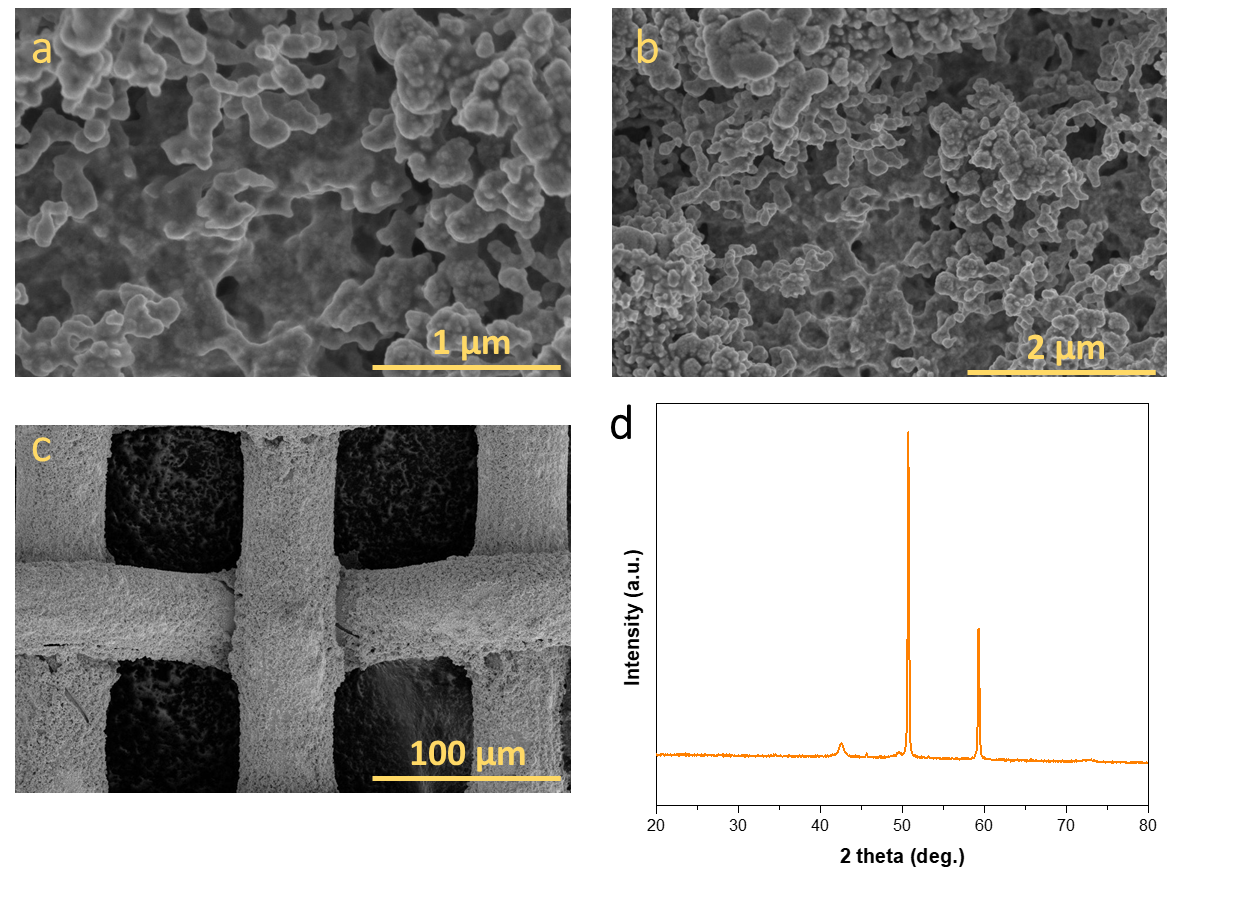


**Figure S16**: Characterization after stability operation for over 200 hours. (a-c) SEM images at different magnifications, and (d) XRD profile of the Cu mesh sample after 200 hours of operation.

**Table S2**. **Model parameters.** Parameters used in the multiphysics model with their corresponding source.

| Symbol | Description | Value | Unit | Reference |
| --- | --- | --- | --- | --- |
| **Catalyst and membrane layer properties** | | | | |
| *s_CL_* | Catalyst layer conductivity | 100 | S/cm | [2] |
| *D_fp_* | Flow plate channel diameter | 0.4 | mm | This work |
| **Transport properties** | | | | |
| *D_CO3_--* | CO_3_^2−^ diffusion coefficient | 0.923×10^-9^ | m^2^/s | [7] |
| *D_HCO3_-* | HCO_3_^−^ diffusion coefficient | 1.185×10^-9^ | m^2^/s |  |
| *D_H+_* | H^+^ diffusion coefficient | 9.311×10^-9^ | m^2^/s |  |
| *D_OH_-* | OH^−^ diffusion coefficient | 5.293×10^-9^ | m^2^/s |  |
| *D_K+_* | K^+^ diffusion coefficient | 1.957×10^-9^ | m^2^/s |  |
| *D_CO2_* | CO_2_ diffusion coefficient | 1.91×10^-9^ | m^2^/s |  |
| **Electrochemical reaction rates** | | | | |
| *i_0,HER_* | Partial exchange current density, HER reaction | 2×10^-6^ | mA/cm^2^ | [1] |
| *i_0,C2_* | Partial exchange current density, 12-electron C_2_ reactions | 5×10^-10^ | mA/cm^2^ | Fit |
| *a_HER_* | Cathodic transfer coefficient, HER reaction | 0.45 |  | [1] |
| *a_C2_* | Cathodic transfer coefficient, 12-electron C_2_ reactions | 1 |  | Fit |
| *g_CO2,C2_* | CO_2_ concentration reaction order, 12-electron C_2_ reactions | 1 |  | Fit |
| *U_C2_* | Equilibrium potential for C_2_ reactions, vs. RHE | 0.07 | V | [8] |
| **Carbonate equilibrium parameters** | | | | |
| *K_1_* | Equilibrium constant, CO_2_+H_2_O « H^+^ + HCO_3_^−^ | 10^−6.37^ | mol/L | [7] |
| *K_2_* | Equilibrium constant, HCO_3_^−^ « H^+^ + CO_3_^2−^ | 10^−10.32^ | mol/L |  |
| *K_3_* | Equilibrium constant, CO_2_ + OH^−^ « HCO_3_^−^ | *K_1_/K_w_* | L/mol |  |
| *K_4_* | Equilibrium constant, HCO_3_^−^ + OH^−^ « H_2_O + CO_3_^2−^ | *K_2_/K_w_* | L/mol |  |
| *K_w_* | Equilibrium constant, H_2_O « H^+^ + OH^−^ | 10^−14^ | mol^2^/L^2^ |  |
| *k_1_* | Forward rate constant, reaction 1 | 3.71×10^-2^ | 1/s |  |
| *k_2_* | Forward rate constant, reaction 2 | 59.44 | 1/s |  |
| *k_3_* | Forward rate constant, reaction 3 | 2.23×10^3^ | L/mol/s |  |
| *k_4_* | Forward rate constant, reaction 4 | 6.0×10^9^ | L/mol/s |  |
| *k_w_* | Forward rate constant, reaction *w* | 1.4×10^-3^ | mol/L/s |  |
| **CO_2_ phase transfer parameters** | | | | |
| *H_CO2_* | Henry’s constant, CO_2_ | 29 | atm/M | [9] |
| *P_g_* | Gas pressure | 1 | atm | Assumed |
| *y_CO2_* | Gaseous CO_2_ mole fraction | 0.01 |  | Assumed |
| *k_MT_* | Mass transfer coefficient, CO_2_ phase transfer | 100 | 1/s | [6] |
| **Bulk electrolyte concentration, 0.1M Nominal KHCO_3_** | | | | |
| *c_CO3_--* | CO_3_^2−^ bulk electrolyte concentration | 9.39×10^-4^ | M | Calculated |
| *c_HCO3_-* | HCO_3_^−^ bulk electrolyte concentration | 0.0979 | M | Calculated |
| *c_H+_* | H^+^ bulk electrolyte concentration | 4.99×10^-9^ | M | Calculated |
| *c_OH_-* | OH^−^ bulk electrolyte concentration | 2.00×10^-6^ | M | Calculated |
| *c_K+_* | K^+^ bulk electrolyte concentration | 0.0998 | M | Calculated |
| *c_CO2_* | CO_2_ bulk electrolyte concentration | 0.0011 | M | [3] |

**References**:

[1] C.A. Obasanjo, G. Gao, J. Crane, V. Golovanova, F.P. García de Arquer, C.-T. Dinh, High-rate and selective conversion of CO2 from aqueous solutions to hydrocarbons, Nature Communications, 14 (2023) 3176.

[2] E.W. Lees, J.C. Bui, D. Song, A.Z. Weber, C.P. Berlinguette, Continuum model to define the chemistry and mass transfer in a bicarbonate electrolyzer, ACS Energy Letters, 7 (2022) 834-842.

[3] X. Min, M.W. Kanan, Pd-catalyzed electrohydrogenation of carbon dioxide to formate: high mass activity at low overpotential and identification of the deactivation pathway, Journal of the American Chemical Society, 137 (2015) 4701-4708.

[4] R. Pärnamäe, S. Mareev, V. Nikonenko, S. Melnikov, N. Sheldeshov, V. Zabolotskii, H. Hamelers, M. Tedesco, Bipolar membranes: A review on principles, latest developments, and applications, Journal of Membrane Science, 617 (2021) 118538.

[5] J.C. Bui, C. Kim, A.Z. Weber, A.T. Bell, Dynamic boundary layer simulation of pulsed CO_2_ electrolysis on a copper catalyst, ACS Energy Letters, 6 (2021) 1181-1188.

[6] R. Kas, K. Yang, G.P. Yewale, A. Crow, T. Burdyny, W.A. Smith, Modeling the local environment within porous electrode during electrochemical reduction of bicarbonate, Industrial & Engineering Chemistry Research, 61 (2022) 10461-10473.

[7] L.-C. Weng, A.T. Bell, A.Z. Weber, Modeling gas-diffusion electrodes for CO_2_ reduction, Physical Chemistry Chemical Physics, 20 (2018) 16973-16984.

[8] L.-C. Weng, A.T. Bell, A.Z. Weber, A systematic analysis of Cu-based membrane-electrode assemblies for CO 2 reduction through multiphysics simulation, Energy & Environmental Science, 13 (2020) 3592-3606.

[9] R.M. Enick, S.M. Klara, CO2 solubility in water and brine under reservoir conditions, Chemical Engineering Communications, 90 (1990) 23-33.

[10] Lee, G., Rasouli, A.S., Lee, B.H., Zhang, J., Xiao, Y.C., Edwards, J.P., Lee, M.G., Jung, E.D., Arabyarmohammadi, F., Liu, H. and Grigioni, I., 2023. CO2 electroreduction to multicarbon products from carbonate capture liquid. Joule, 7(6), pp.1277-1288.

[11] Song, H., Fernández, C.A., Venkataraman, A., Brandão, V.D., Dhingra, S.S., Arora, S.S., Bhargava, S.S., Villa, C.M., Sievers, C., Nair, S. and Hatzell, M.C., 2024. Ethylene Production from Carbonate Using a Bipolar Membrane Electrolysis System. ACS Applied Energy Materials.

[12] Lee, J., Liu, H. and Li, W., 2022. Bicarbonate Electroreduction to Multicarbon Products Enabled by Cu/Ag Bilayer Electrodes and Tailored Microenviroments. ChemSusChem, 15(22), p.e202201329.

[13] Wang, J., Zhang, Z., Wu, W., Liu, Y., Dong, B., Wang, Y. and Wang, Y., 2023. Unraveling How Local Environments Impact Multicarbon Product Electrosynthesis in Active Carbon Solutions. ACS Energy Letters, 9(1), pp.110-117.
